# Robustness of sex-differences in functional connectivity over time in middle-aged marmosets

**DOI:** 10.1038/s41598-020-73811-9

**Published:** 2020-10-06

**Authors:** Benjamin C. Nephew, Marcelo Febo, Ryan Cali, Kathryn P. Workman, Laurellee Payne, Constance M. Moore, Jean A. King, Agnès Lacreuse

**Affiliations:** 1 Department of Biology and Biotechnology, Worcester Polytechnic Institute, Worcester, MA 01609 USA; 2 Center for Comparative Neuroimaging, University of Massachusetts Medical School, Worcester, MA 01655 USA; 3 Department of Psychiatry, University of Florida, Gainesville, FL 32610 USA; 4 Department of Psychiatry, University of Massachusetts Medical School, Worcester, MA 01655 USA; 5 Psychological and Brain Sciences, University of Massachusetts, Amherst, MA 01003 USA; 6 Neuroscience and Behavior Program, University of Massachusetts, Amherst, MA 01003 USA; 7 Center for Neuroendocrine Studies, University of Massachusetts, Amherst, MA 01003 USA

**Keywords:** Cognitive ageing, Cognitive neuroscience, Neural ageing, Neural circuits, Functional magnetic resonance imaging

## Abstract

Nonhuman primates (NHPs) are an essential research model for gaining a comprehensive understanding of the neural mechanisms of neurocognitive aging in our own species. In the present study, we used resting state functional connectivity (rsFC) to investigate the relationship between prefrontal cortical and striatal neural interactions, and cognitive flexibility, in unanaesthetized common marmosets (*Callithrix jacchus*) at two time points during late middle age (8 months apart, similar to a span of 5–6 years in humans). Based on our previous findings, we also determine the reproducibility of connectivity measures over the course of 8 months, particularly previously observed sex differences in rsFC. Male marmosets exhibited remarkably similar patterns of stronger functional connectivity relative to females and greater cognitive flexibility between the two imaging time points. Network analysis revealed that the consistent sex differences in connectivity and related cognitive associations were characterized by greater node strength and/or degree values in several prefrontal, premotor and temporal regions, as well as stronger intra PFC connectivity, in males compared to females. The current study supports the existence of robust sex differences in prefrontal and striatal resting state networks that may contribute to differences in cognitive function and offers insight on the neural systems that may be compromised in cognitive aging and age-related conditions such as mild cognitive impairment and Alzheimer’s disease.

## Introduction

Studying nonhuman primates (NHPs) is essential to obtaining a comprehensive understanding of neurocognitive aging in our own species. The common marmoset (*Callithrix jacchus*) is a small-bodied New World monkey (300–500 g) which is emerging as an important model for human aging. As with other primates, it shares many aspects of brain organization and cognitive and social processes with humans^1^, but has the unique advantage of a relatively short life expectancy (approximate mean of 10 years), making it ideally suited for longitudinal studies^2^. Sensory and neurodegenerative changes in marmosets appear between 7–10 years of age^2^, with response strategy deficits in cognition apparent at 4 years and inhibitory control deficits at appearing at 7–8 years^3^. Longitudinal investigations have been difficult to implement in aging NHPs^4^ but are critical for understanding cognitive change and associated factors within the same individual^5^.

As part of an ongoing study, Lacreuse and colleagues collected yearly behavioral, physiological, cognitive and neuroimaging data in a cohort of male and female marmosets that were 5–6 years old at study onset^6–9^. The initial reports revealed large and robust sex differences in reversal learning performance. In reversal learning, monkeys have to select the rewarded stimulus in pairs of stimuli (discrimination) and reverse their response as the previsouly unrewarded stimulus becomes the rewarded stimulus (reversal). This test evaluates cognitive flexibility, which can be assessed by recording the number of trials monkeys need to perform a reversal relative to a discrimination. It was determined that females required consistently more trials than males to perform reversals relative to initial discrimination. In addition, these cognitive differences are strongly correlated with sex differences in resting-state functional connectivity (rsFC) assessed using magnetic resonance imaging (MRI)^9^.

In the current study, rsFC of male and female common marmosets evaluated in LaClair et al. 2019 were examined 8 months (comparable to a 5–6 year interval in humans given similar aging trajectories across primates^10^) following the initial assessment of cognition and functional brain connectivity to investigate sex differences and potential changes over time. The deficiency in female cognitive flexibility, which correlated with decreased cognitive flexibility in females at the initial time point^9^, was consistent over this time period^6^. The objectives of the present investigation were to identify and characterize potential changes and sex differences in neural connectivity over 8 months and evaluate associations between functional connectivity and cognitive flexibility. Based on the consistency in cognitive performance across time, it was hypothesized that differences in rsFC would be similar as well.

## Results

### Resting state functional connectivity

**Figure 1:**
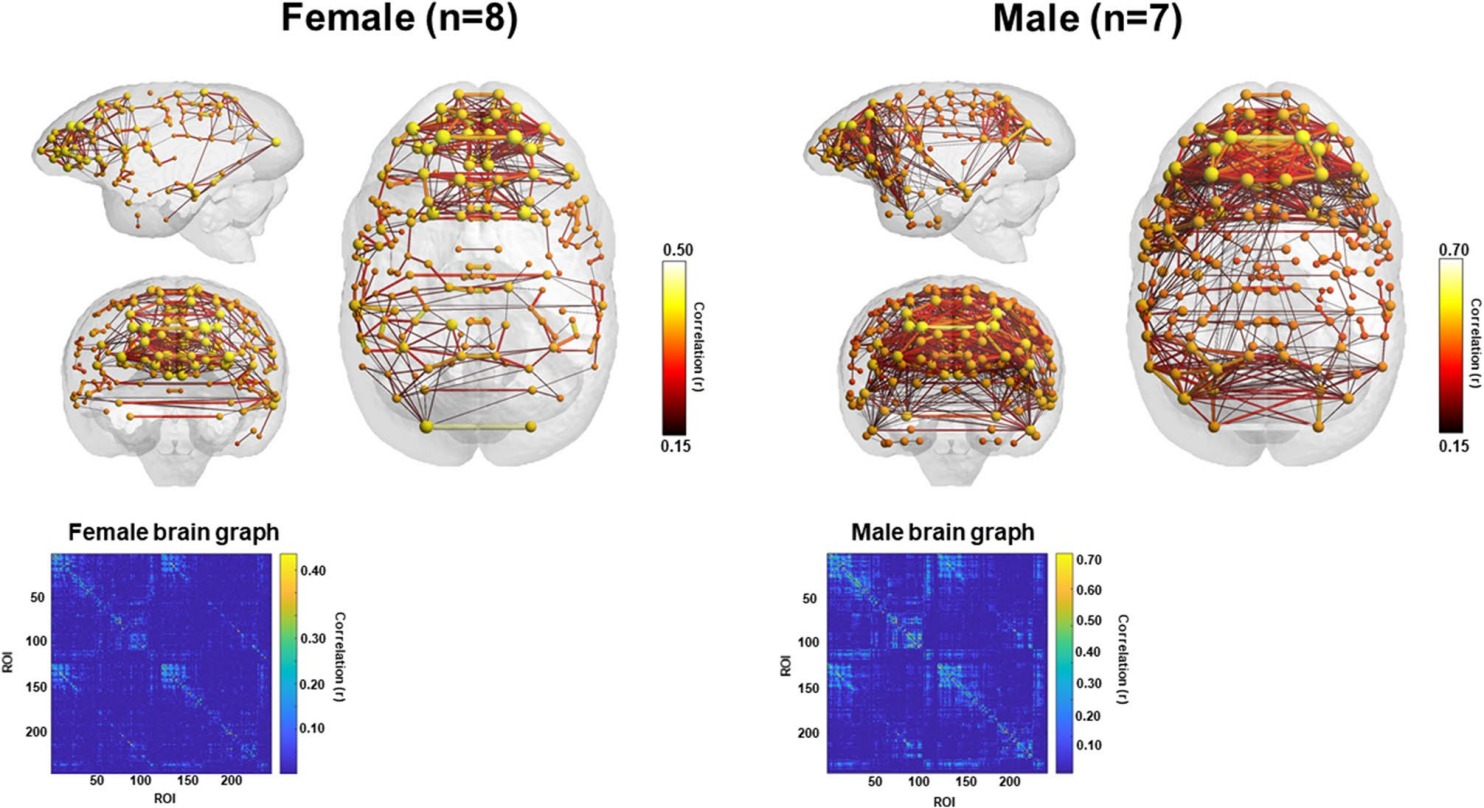
3D functional network maps of the marmoset monkey brain display stronger clustering in males compared to females. Figures display sagittal, coronal, and axial planes with nodes (spheres) and edges (lines connected to spheres) overlaid onto a 3D atlas. Maps were thresholded at z > 0.15 and represent the top 10% of connections (density k = 0.10). For all pairwise correlations, a density threshold of 10% corresponds to about a lower bounds r value of approximately 0.2 (with highest values of approximately 0.7–0.8).

**Figure 2:**
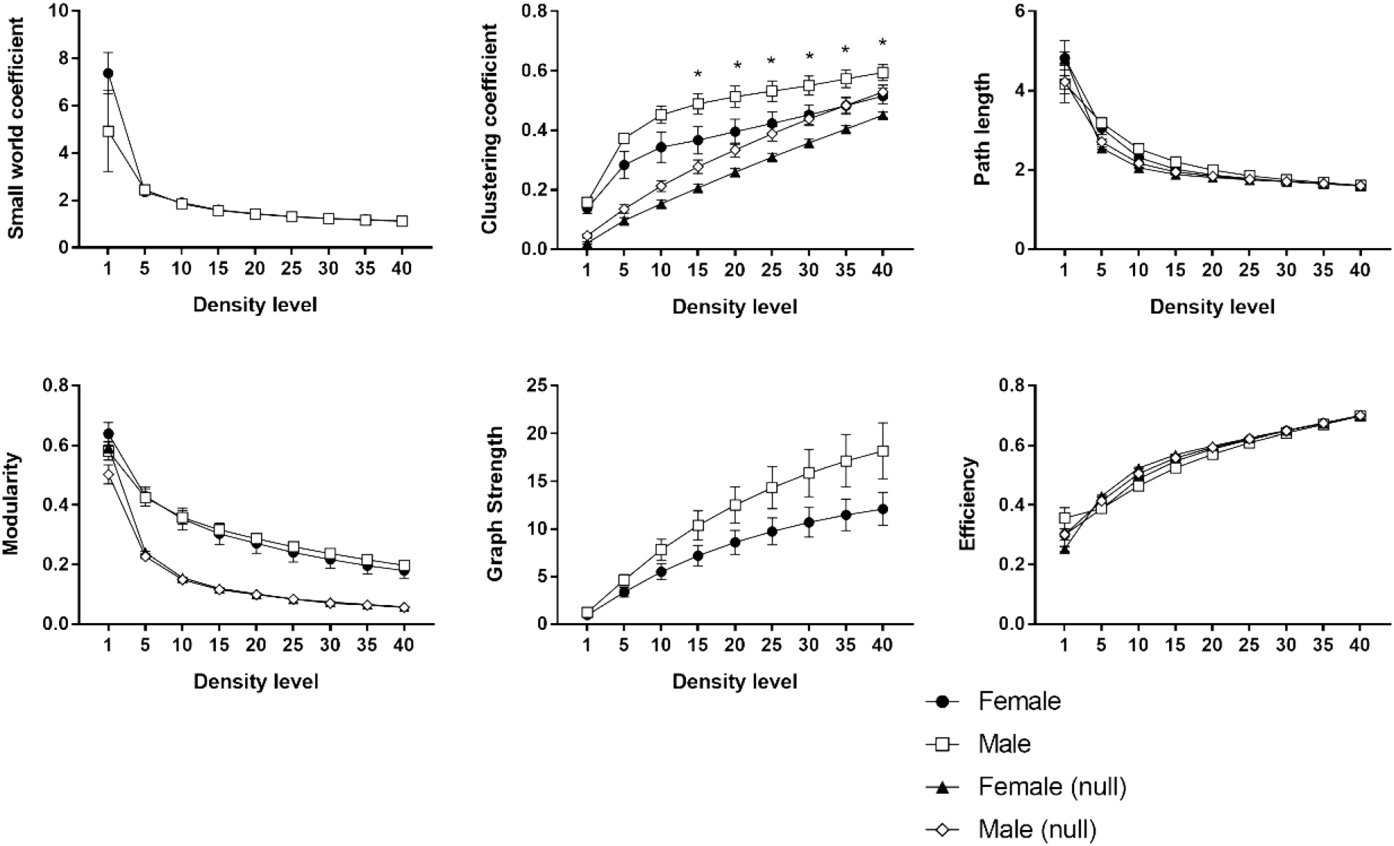
Stronger clustering coefficient was observed in males compared to females (p = 0.05, t-test). Empty circles and filled squares indicate the identical metrics alternatively calculated for random networks with the same density and edge weight.

**Figure 3:**
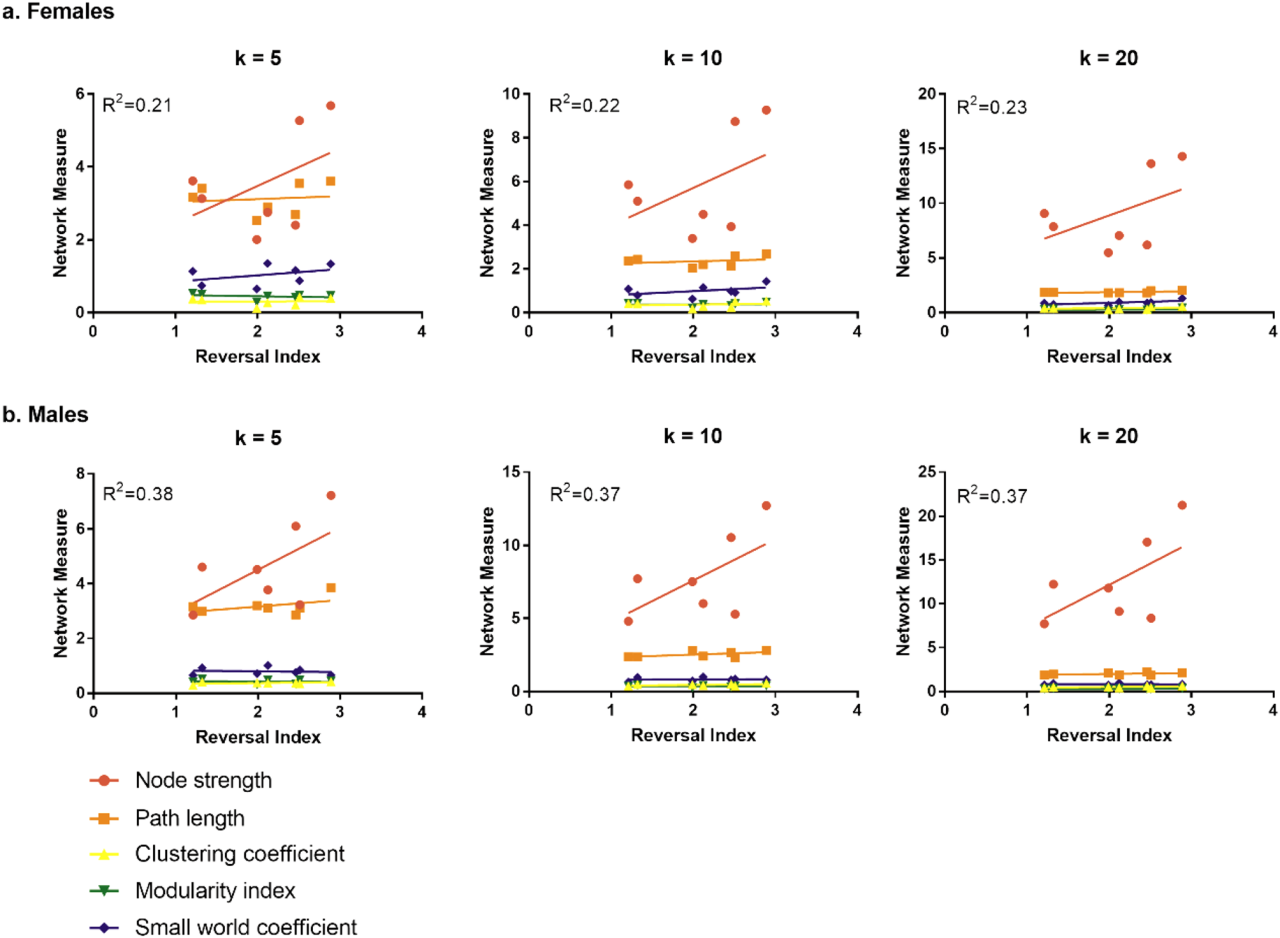
There was a trend for global node strength to be positively correlated with poorer cognitive performance, as measured by a greater reversal index, in both females **(a)** and males **(b)**.

**Figure 4:**
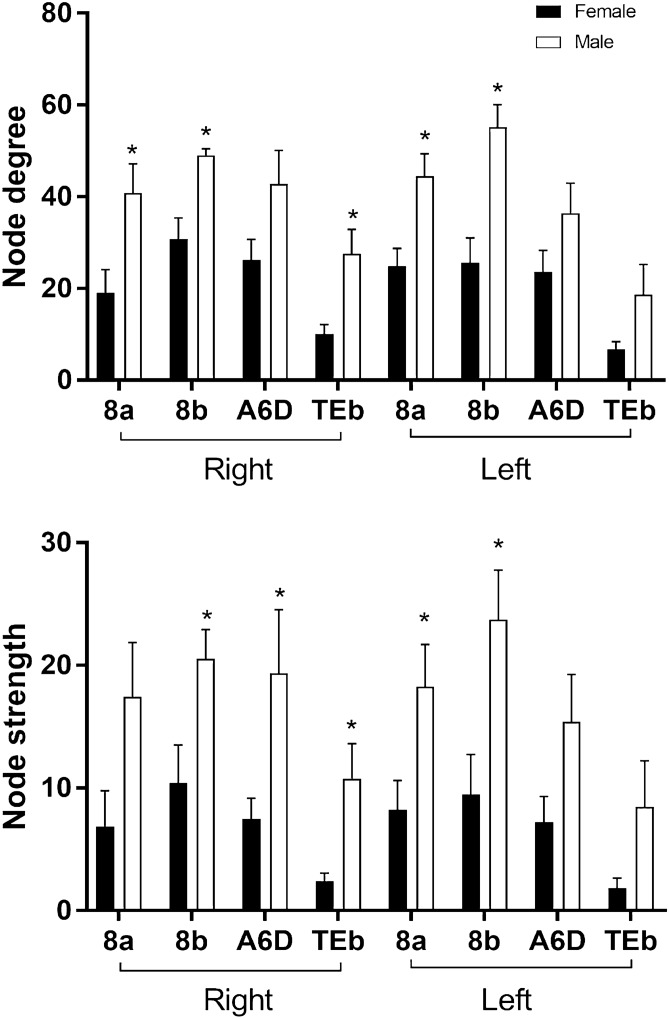
Using seed regions in the following locations: 8a, 8b, dorsolateral prefrontal cortex, A6D, premotor cortex and TEb, sex differences in brain connectivity metrics were observed in lateral and inferior temporal cortical regions. Both node degree and node strength were greater in males than females across these regions. * denotes significant sex differences (p < 0.05, t-test).

Fifteen animals (7 females, mean age = 6.75 years, SD = 0.73; 8 males, mean age = 6.88 year, SD = 0.81) were imaged on a day within their period of cognitive testing. Averaged three-dimensional (3D) functional network maps of the marmoset monkey brain revealed functional connectivity between a greater number of regions in males relative to females (Fig. 1). The 3D maps illustrate symmetrical nodal interactions both across and within brain hemispheres, which had a greater total number of edges in male vs females (Fig. 1). Consistent with greater nodal interactions observed in males (Fig. 1), there was greater clustering coefficient (k15–40 p = 0.05) and a trend for greater graph strength (k30–40 p = 0.1), in males compared to females (Fig. 2). This indicates a greater number of strong functional ties between brain regions in males than females. No sex differences were observed in the small world coefficient, modularity index, or efficiency. There was a non-significant trend for node strength correlating with the reversal index (RI) in both females and males, with a greater RI reflecting poorer reversal performance (Fig. 3). Analysis of node strength of individuals regions indicated significantly greater node strength values in males than females in ventrolateral prefrontal cortical (VLPFC) area 8 (right 8b, p = 0.02; left 8a p = 0.03 and left 8b p = 0.02), premotor cortical area 6 (right A6D p = 0.04), and inferior temporal area b (right TEb p = 0.009) (Fig. 4). Similarly, node degree was greater in male *vs.* females in VLPFC area 8 (right 8a p = 0.02; right 8b p = 0.003; left 8a p = 0.007; left 8b p = 0.001), and inferior temporal area b (right TEb p = 0.006).

**Figure 5:**
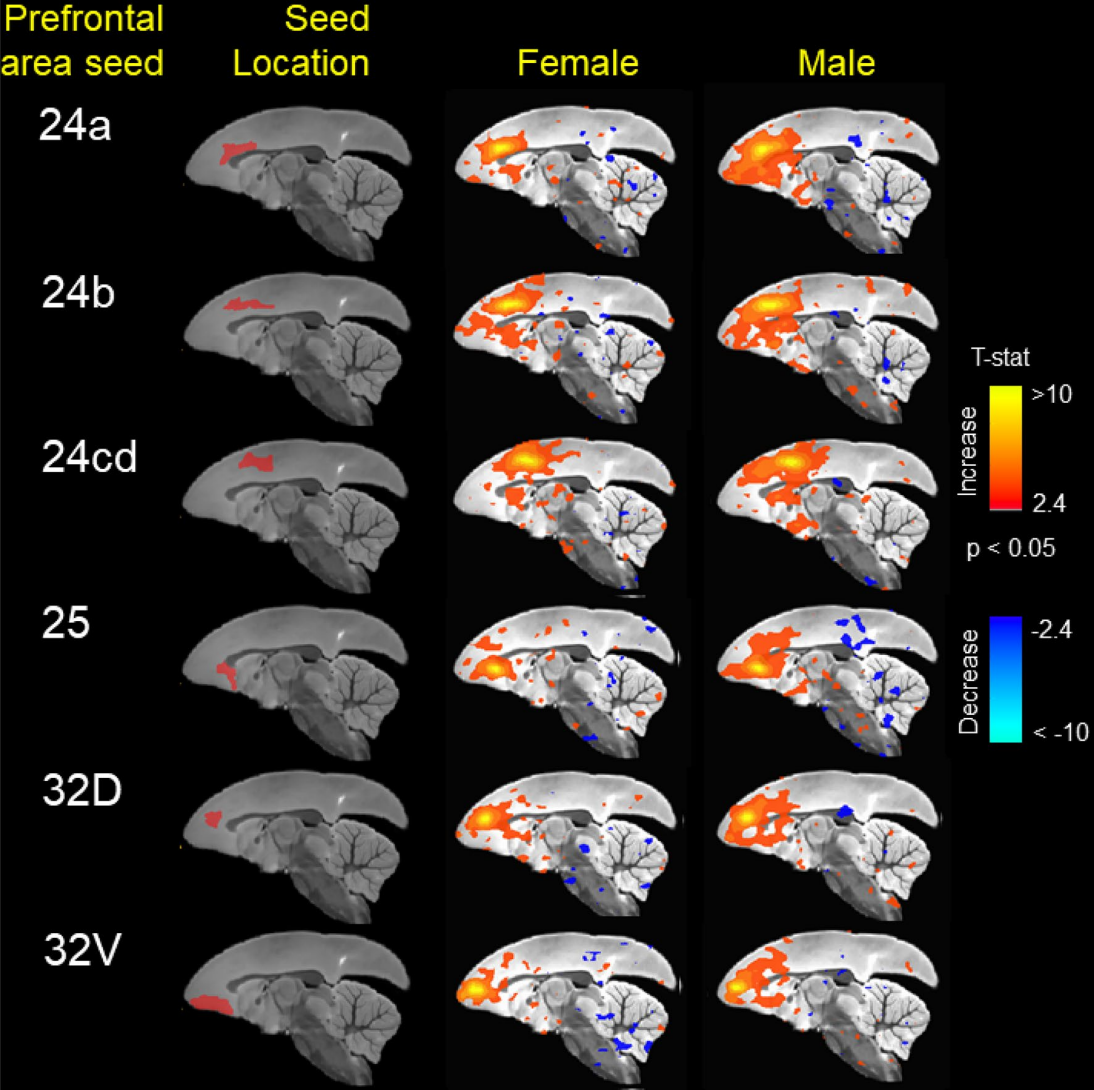
Seed based functional connectivity in various medial prefrontal cortex subdivisions indicates that males have greater functional connectivity with these regions than females. Maps represent mean functional connectivity across all animals within each group, thresholded by statistical t values (t > 2.3, p < 0.05).

**Figure 6:**
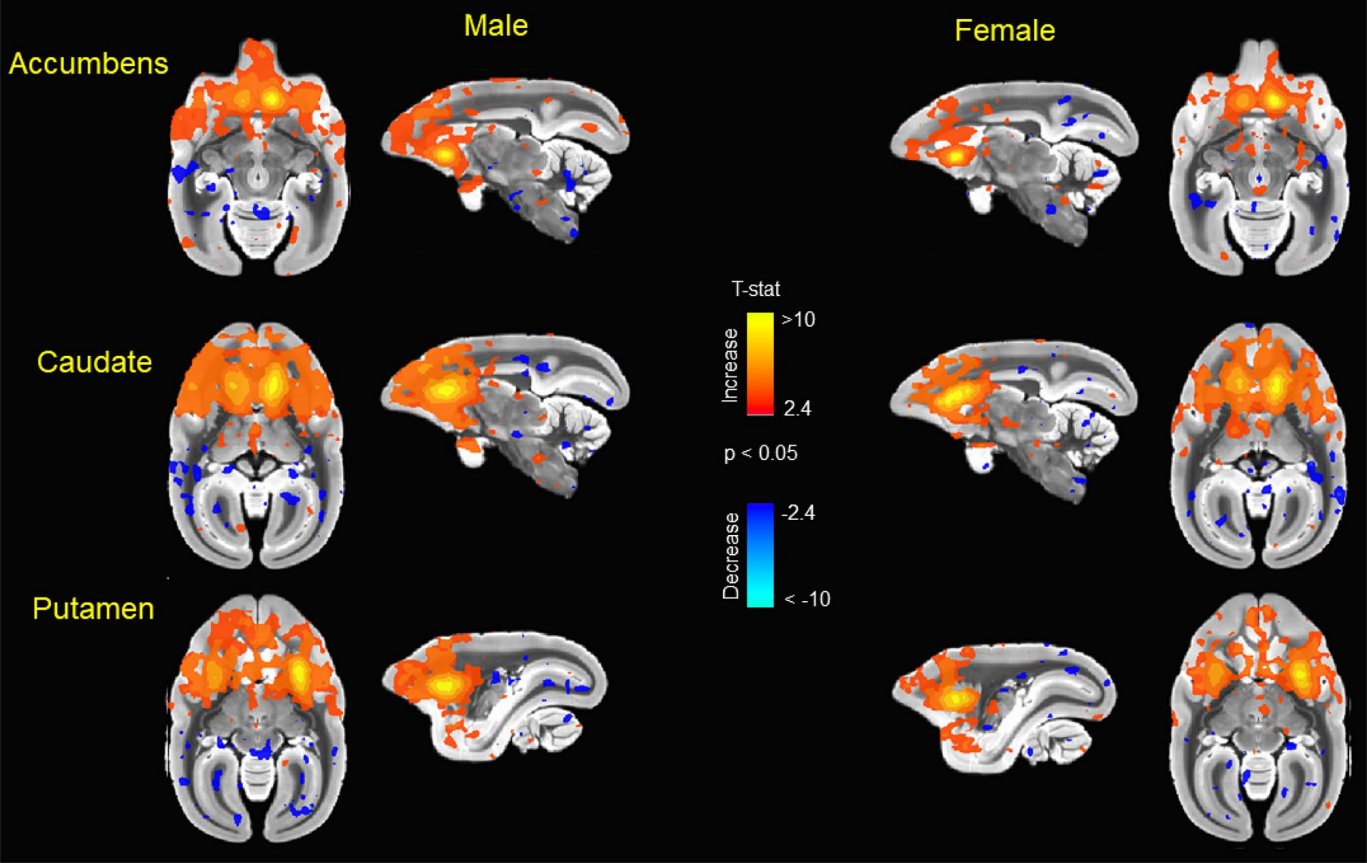
Seed based functional connectivity in nucleus accumbens, caudate nucleus, and putamen indicates that males have greater functional connectivity with these regions than females. Maps represent mean functional connectivity across all animals within each group, thresholded by statistical t values (t > 2.3, p < 0.05).

**Figure 7:**
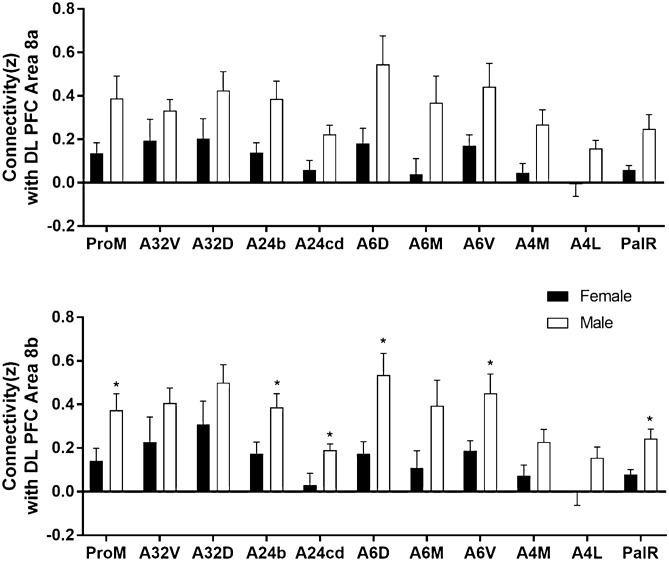
Medial prefrontal cortex regions with stronger connectivity to the dlPFC (8a and 8b) in males compared to females. ProM: Ventrolateral Prefrontal Cortex (Proisocortical motor region), A32V: Medial Prefrontal Cortex (Area 32 ventral), A32D: Medial Prefrontal Cortex (Area 32 dorsal), A24b: Medial Prefrontal Cortex (Area 24b), A24cd: Medial Prefrontal Cortex (Area 24 cd), A6D: Motor and Premotor Cortical Regions (Area 6 Dorsal), A6M: Motor and Premotor Cortical Regions (Area 6 Medial), A6V: Motor and Premotor Cortical Regions (Area 6 Ventral), A4M: Motor and Premotor Cortical Regions (Area 4 Medial), A4L: Motor and Premotor Cortical Regions (Area 4 Lateral),PalR: Insula and others in Lateral Sulcus: Parainsular Cortex Rostral. * denotes a significant difference (p < 0.05, t-test).

**Figure 8:**
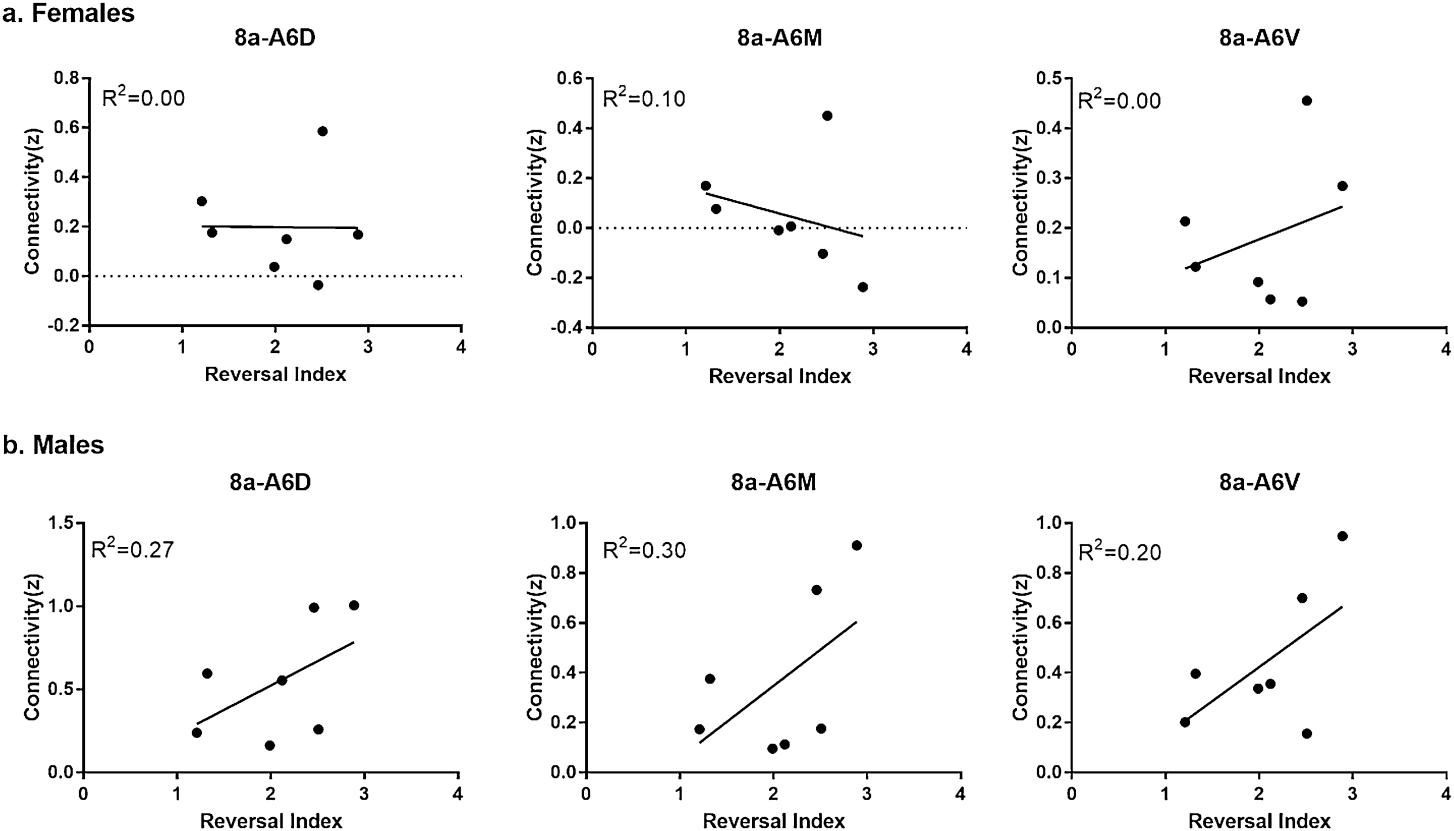
Connectivity between seed regions located in the dlPFC (8a) and motor and premotor cortical regions (A6D, A6M, A6V) was positively correlated with reversal index in males only.

**Figure 9:**
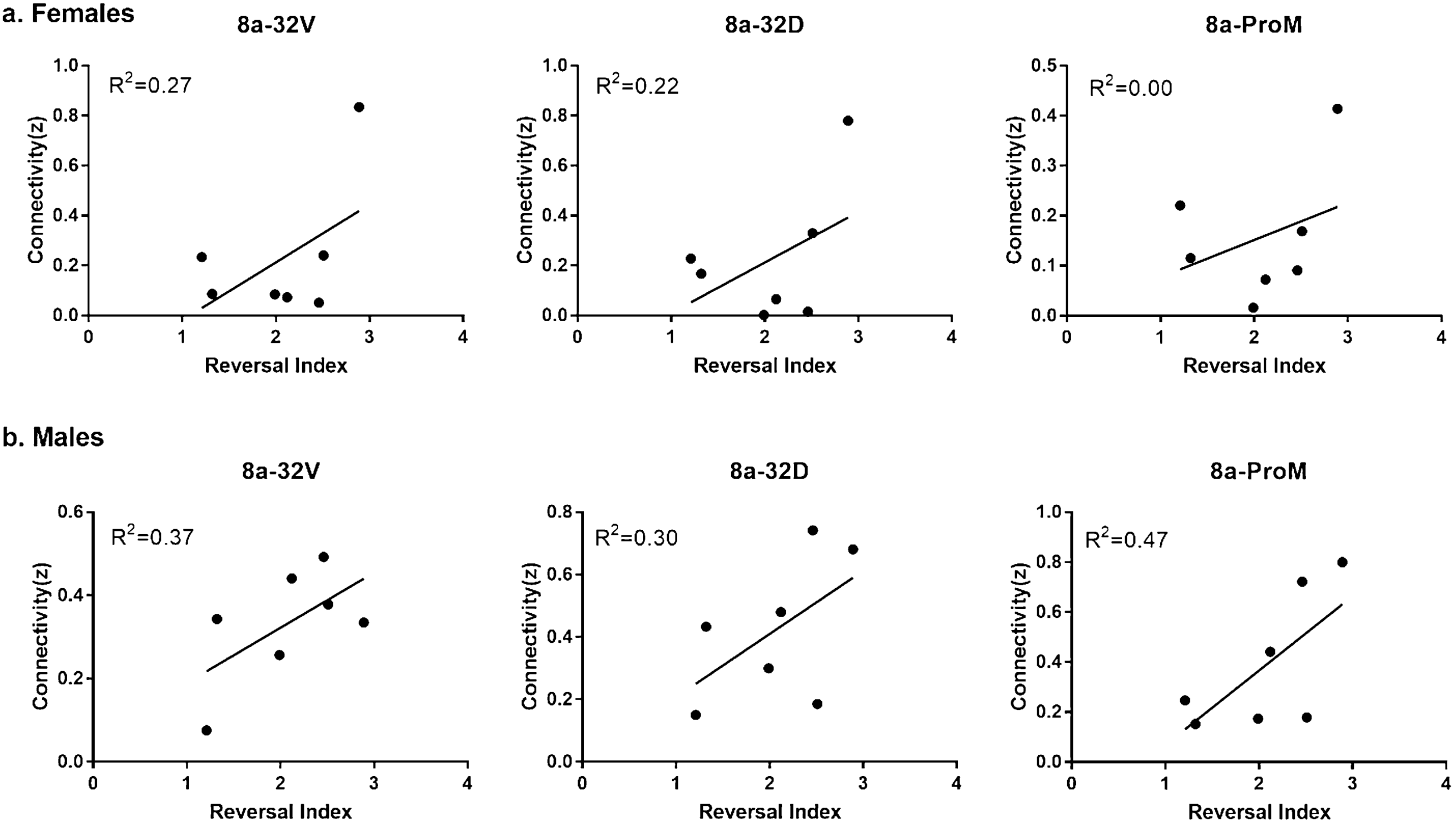
Connectivity between seed regions located in the dlPFC (8a) and medial PFC as well as the ventrolateral PFC (A32V, A32D, ProM) were correlated with reversal index in both males and females.

In addition to network connectivity metrics, seed based functional connectivity was also analyzed. Figure 5 displays maps of functional connectivity with prefrontal cortical subregions 24a, 24b, 24cd, 25, 32D and 32V. Figure 6 displays maps of functional connectivity with the nucleus accumbens, caudate and putamen nuclei. The maps were relatively consistent between male and female marmosets and were also remarkably similar to our previous results for data collected approximately 8 months earlier in the same animals^9^. Due to the sex differences in node strength observed in PFC area 8 and premotor area 6, functional connectivity was assessed between these regions and other areas of the PFC and premotor/motor cortices (Fig. 7). A trend towards greater functional connectivity between area 8a and several cortical regions in males than in females was observed. The VLPFC area 8b had greater functional connectivity with the proisocortical motor region (p = 0.02), medial prefrontal areas A24b (p = 0.02) and A24cd (p = 0.02), premotor cortical areas A6D (p = 0.006) and A6V (p = 0.01), and the rostral parainsular cortex (p = 0.004). A positive correlation between the reversal index and VL-premotor cortex functional connectivity was observed in males only, with R^2^ values of 0–0.10 for females and 0.20–0.30 for males (Fig. 8). Functional connectivity between the VL and medial PFC was positively correlated with the reversal index in both male and females, with R^2^ values of 0.22 -0.47 (Fig. 9).

**Figure 10:**
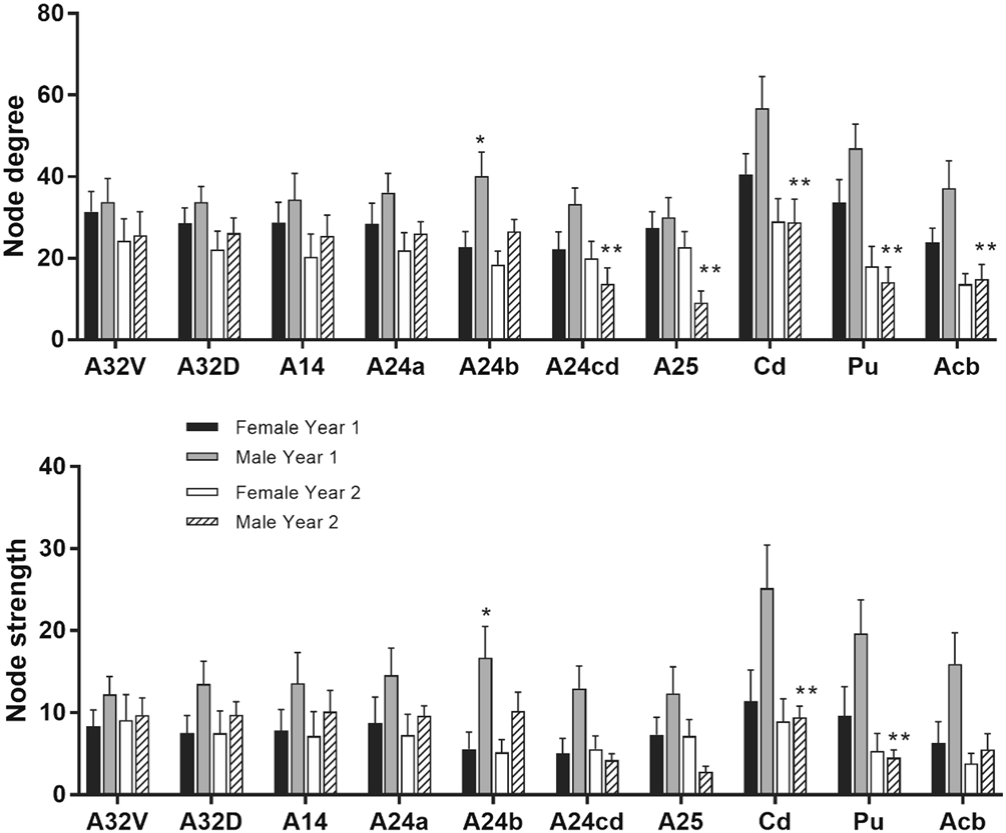
When comparing with scan 1 data, a significant decrease in node degree was observed in medial prefrontal cortex (A24cd), A25, caudate, putamen, and accumbens at scan 2 only in males. Similarly, there were significant effects of time on node strength in the caudate and putamen only in males. The greater node degree and strength in the A24b at scan 1 was not significant at scan 2. * denotes a significant sex difference. ** denotes significant effect of time.

**Figure 11:**
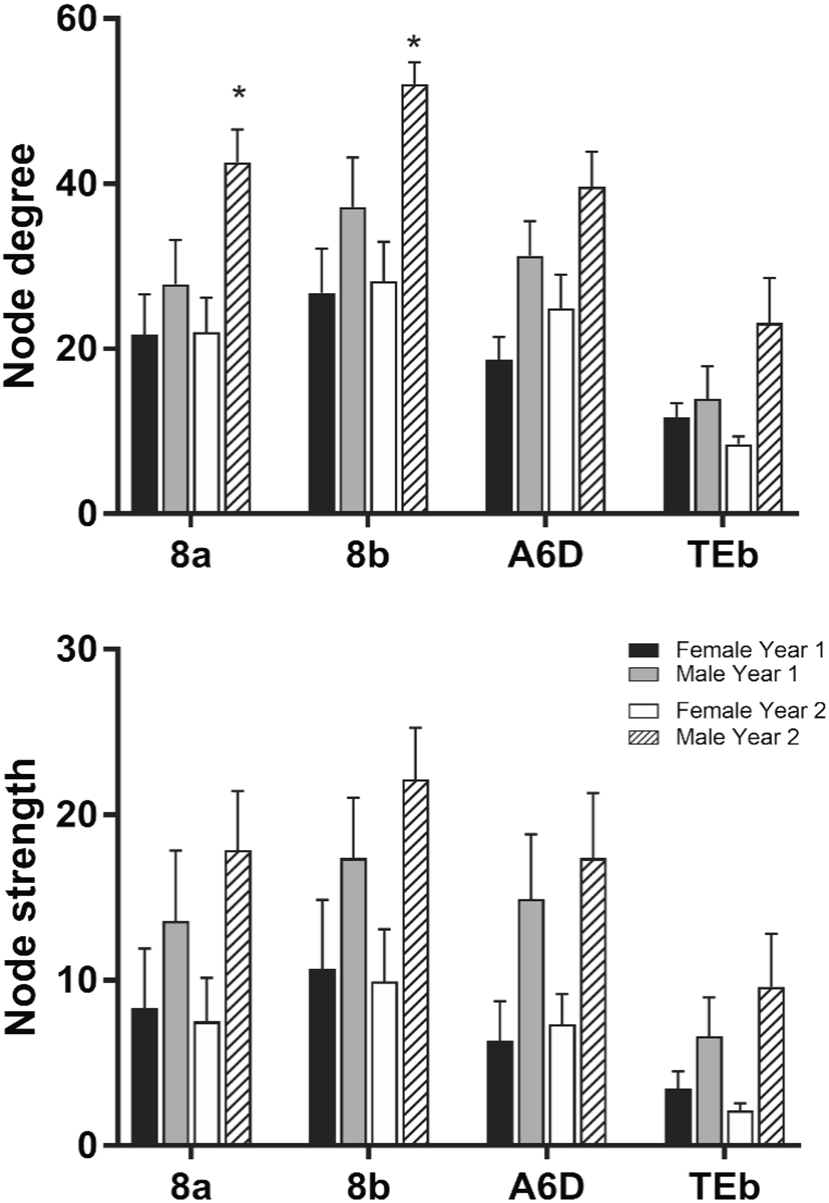
Time-specific sex differences were observed in node degree in VLPFC areas (8a, 8b), with males having significantly greater node degree at time 2 (p < 0.05). * denotes a significant sex difference.

As indicated above, the late middle-age marmosets in the present study were scanned twice, with 8 months between scans (similar to a 5–6 year interval in humans). Network metrics and seed-based functional connectivity results between the two scans were compared. Supplemental Fig. 1 shows a high consistency for modularity, path length, clustering coefficient and graph strength for the two time points in both male and female marmosets. However, a detailed look at specific regions of interest revealed differences between time 1 and time 2 that varied with sex. When comparing with scan 1 data, a significant decrease in node degree was observed in medial prefrontal cortex (A24cd), A25, caudate, putamen, and accumbens at scan 2 only in males. Similarly, there were significant effects of time on node strength in the caudate and putamen only in males. The greater node degree and strength in the A24b at scan 1 was not significant at scan 2 (Fig. 10). Sex differences in node degree were observed in VLPFC areas 8a (main effect of sex F_1,29_ = 7.8, p = 0.008) and 8b (main effect of sex F_1,29_ = 11.1, p = 0.002) at time 2 and not time 1 (Fig. 11).

## Discussion

In a valuable and rare replication of our initial study^9^, male marmosets exhibited stronger functional connectivity relative to females 8 months later, paralleling the cognitive results from these animals, where males exhibit greater cognitive flexibility at both time points^6^, similar to reports in male child^11^ and adult humans^12^. There were limited effects of time. The consistent sex differences and related cognitive associations were characterized by greater node strength and/or degree in several prefrontal, premotor and temporal regions, as well as stronger intra PFC connectivity in males. Specific time-dependent differences in connectivity were identified in the mPFC, caudate, putamen, and accumbens. These findings are regionally consistent with studies in humans^13–18^, further validating the use of NHPs in the study of neural mechanisms of sex differences and cognitive aging. Despite recent improvements in fMRI study replication^19^, inconsistency in replication is a common issue^20,21^, as well as in science in general^22^. Although increasing sample sizes enhances reproducibility and the current sample sizes are modest, the essential factors for the consistency of the cognition and fMRI results in the present study are likely the robust differences in cognition and FC and consistency in behavioral data collection, husbandry, and imaging acquisition and analytical methodology.

Much of the previous work on sex differences in the human brain have focused on anatomical features, such as volume, surface area, and white matter track attributes^23–26^. However, there is growing evidence of sex differences in functional connectivity as well^23,25,27–29^. A recent machine learning approach was able to accurately classify sex based on rsFC, with many of the most substantial differences in the frontal regions^28^, supporting related earlier studies of neural sex differences^30,31^. Interestingly, recent studies suggest that sex differences in functional connectivity, particularly in frontal areas, may be at least in part due to fluctuations of resting state activity with the menstrual cycle in females^28,32,33^. The presence of sex differences in resting state networks in human subjects is somewhat unclear and differences and the direction of these differences can vary according to age, specific task used, and disease state. Some studies report differences in the networks that exhibit high functional connectivity in women and men^34^, and men have higher functional connectivity between cognitive and sensorimotor regions than women^34^. In contrast, others have reported reduced functional connectivity across various networks in males versus females^35^, or no differences^36^, with more recent investigations documenting sex differences. In one uniquely robust study, several resting state data sets were used to determine brain region classifiers for sex differences and these included regions such as the cingulate cortex and other limbic and prefrontal cortical regions^37^. This is consistent with the regions noted in the present study as being significantly different between male and female marmosets. However, there is a clear need for additional study to characterize sex-associated differences in cognition and functional connectivity in detail.

In the marmoset, robust node-related sex differences in PFC, premotor, and temporal regions were documented. Global PFC activity is critical for overall cognitive performance^38^, and acute increases in connectivity in this region during cognitive challenges have also been documented^39^, including tasks directly involving inhibitory control^40^, a critical component of the reversal learning task. The stronger node strength and degree in male marmoset PFC compared to females may mediate the male advantage in reversal learning through improved inhibitory control, allowing males to adapt more quickly to reversals during the reversal learning task.

Males also exhibited stronger connectivity between premotor region 6 and cognition- related PFC areas, which could have contributed to the enhanced reversal learning performance. Premotor areas have been implicated in cognition^41,42^ and suggested to be a gateway between cognitive and motor areas^43,44^. Greater gray matter volume in the premotor cortex has been observed in human males^45^. While it is unknown if the rsFC differences in the current marmosets are associated with corresponding greater gray matter volume, this is a potential explanation for the greater premotor connectivity and associated cognitive advantage in males.

Although the temporal cortex is not a common focus in cognition research, it has been implicated in reversal learning in rats^46^, visual based tests of semantic memory^47^, and pathological changes in protein levels in patients afflicted with mild cognitive impairment^48^. Sex differences in the activation of the temporal cortex have been observed in studies of the visual processing of motion^49^, and sex specific lateralization of temporal lobe activity has been reported in response to both verbal and spatial tasks^50^, similar to the lateralization in the current marmoset data. It is possible that the sex dependent role of the temporal cortex could become more substantial over time and/or with age related cognitive pathologies.

One specific group of connectivity associations unique to the male marmosets were the positive dlPFC motor area correlations with cognitive performance. While traditionally known for motor related functions, premotor areas have been increasingly implicated in cognition^51–53^. Neural projections from the premotor cortex may be particularly essential for reward based tasks requiring discrimination^53^, where this region functions to encode task dependent responses following training. In the current marmoset RI data, the involvement of motor and premotor areas during training and testing may have led to improved cognitive flexibility in males, potentially through the integration of cognitive and physical control mechanisms^54^. Data on sex differences in motor region connectivity is limited, but greater functional connectivity has been observed in somatomotor regions, as well as the PFC, in a healthy group compared to patients suffering from mild cognitive impairment^55^, indicating that FC in this region could be a marker of healthy aging.

The ROI based results directly support the node strength and degree findings, indicating a consistent pattern of stronger connectivity within the PFC and between the PFC and motor cortex in male marmosets. Since the ROI’s were selected due to their significance in network sex differences in marmosets, these results also validate the network measures, which offer the advantages of greater global significance when compared to ROI data between a handful of regions. Although network measures are derived from regional data, the two are not necessarily equivalent with regards to significant differences between groups. Taken together, the present marmoset node strength and degree and ROI connectivity data support studies of region specific neural sex differences in humans, underscoring their value as model for human neuroplasticity across the lifespan.

Both increases and decreases in FC have been associated with aging in humans. Early studies of the default mode network (DMN) in humans reported minor sex and age differences in the connectivity within this network^56^. Later reports described decreased FC with age, with sex differences in local connectivity patterns^57^, and robust interactions between sex and age^58^. For instance, both men and women showed decreased connectivity with age in the DMN, with men showing a steeper slope of decline. However, in other networks, such as the fronto-parietal network, males and females showed divergent connectivity trajectories with age, with males showing increased connectivity and females showing decreased connectivity with age.

There were several male-specific decreases in node degree and/or strength over time in the present group of marmosets, including key nodes identified in prior marmoset imaging studies^59^, such as the medial prefrontal cortex (A24cd), A25, caudate, putamen, and accumbens. There were also increases in functional connectivity in the vlPFC of males relative to females, which has been specifically implicated in primate reversal learning^60,61^. Lesions of this area increase anxiety and perseverative behavior and mediate the regulation of negative emotion^62,63^. Studies of the vlPFC in primates is considered essential to progress in understanding the neurobiological mechanisms for the cognitive and emotional symptoms of a range of psychiatric maladies^64^. Due to the consistent patterns of sex differences in MCI and AD, the vlPFC merits increased focus in subsequent investigations of the effects of time and/or sex on primate cognition.

Limitations to the present study include the use of late middle aged marmosets and a relatively short time interval between scans. Due to the logistical challenges of scheduling the fMRI scans and the age range of the population, another limitation to the longitudinal aspect of the study is that there was overlap in the age ranges of the marmosets at scan 1 and scan 2. Future studies should expand on the longitudinal aspects through the use of both younger and older time points and associated extended intervals between assessments. In addition, primate neurocognitive aging studies would benefit greatly from ICA based analysis of functional connectivity and the use of multiple cognitive assessments to determine if changes are dependent on the task and/or type of cognition.

In conclusion, rsFC analyses revealed substantially stronger neural connectivity in male marmosets relative to females, with differences in overall network metrics, overall node metrics, and/or regional metrics in the PFC, premotor area, temporal cortex, caudate, putamen, and nucleus accumbens. Sex-dependent correlations between reversal learning and neural connectivity measures suggest that sex-dependent patterns of connectivity may contribute to the sex difference in reversal learning. These results are highly consistent with data from these same animals when they were assessed 8 months prior, as well as human neuroimaging data, supporting the hypothesis that sex differences in cognitive performance have identifiable intrinsic neural correlates^65^. An improved understanding of sex dependent neural mechanisms of cognitive aging will enhance research on targeted preventative measures and interventions for age-related pathologies such as mild cognitive impairment and AD.

## Methods

### Subjects

The animals were cared for in accordance with the guidelines published in the Guide for the Care and Use of Laboratory Animals, 8th edition (2011). The studies were approved by the Institutional Animal Care and Use Committee of the University of Massachusetts Amherst and the University of Massachusetts Medical School, Worcester. The parent study included 28 marmosets ranging from four to six years old (14 females, 14 males). From these, 18 monkeys with cognitive data (9 females and 9 males, mean age = 6.12, SD = 0.65) were imaged at Time 1, as reported in LaClair et al. (2019). The current study includes 15 monkeys from this dataset, 7 females and 8 males (mean age = 6.85, SD = 0.73), re-scanned approximately 8 months later (Time 2; see Table 1), which corresponds to a 4–5 year period in humans. All marmosets were housed in male/female pairs at the University of Massachusetts, Amherst and maintained under a 12/12 h light/dark cycle (lights on at 7:30 A.M.) at an ambient temperature of 80° F with a relative humidity of 50%. The pairs were housed in steel mesh cages (101 cm × 76.2 cm × 78.7 cm) equipped with perches, hammock, nest boxes, and branches to encourage species-typical behaviors. Male marmosets were vasectomized in adulthood, before the start of the study, to avoid pregnancy. The characteristics of the marmosets and the tests they performed can be seen in Table 1. The monkeys were fed a daily diet of fresh food including fruits, vegetables, nuts and seeds, various breads, and ZuPreem marmoset food. Fruit and nuts were provided twice daily up until 2 h before and immediately after cognitive testing and water was available ad libitum. The monkeys were provided with daily enrichment, including foraging tubes and a variety of toys.

**Table 1:** Marmoset DOB, ages at cognitive testing and imaging, imaging date, and interval between fMRI scans 1 and 2 in years.

Sex	DOB	Imaging date	Cognitive testing age	Imaging age	fMRI interval
Male	6/1/2011	12/5/2017	6.53	6.52	0.70
Male	6/18/2010	12/8/2017	7.40	7.48	0.67
Male	5/1/2011	10/23/2017	6.37	6.48	0.67
Male	9/3/2009	12/21/2017	8.12	8.30	0.65
Male	8/20/2010	12/18/2017	7.12	7.33	0.53
Male	10/28/2010	10/23/2017	6.88	6.99	0.84
Male	5/13/2012	2/9/2018	5.33	5.75	0.62
Male	4/28/2012	1/12/2018	5.38	5.71	0.59
Female	9/16/2010	12/8/2017	7.13	7.23	0.67
Female	7/5/2011	10/23/2017	6.19	6.31	0.67
Female	1/4/2010	12/21/2017	7.75	7.97	0.65
Female	7/5/2011	12/18/2017	6.31	6.46	0.53
Female	3/22/2011	10/10/2017	6.48	6.56	0.80
Female	1/18/2011	2/9/2018	6.71	7.07	0.62
Female	11/9/2011	1/12/2018	5.84	6.18	0.59

### Overall experimental description

Monkeys received comprehensive assessments of cognitive function, stress reactivity and motor function. The details regarding each assessment are provided in LaClair et al. 2019^9^. Monkeys performed cognitive tasks 5 days per week, with completion of the reversal learning task typically spanning 2 or more months. Tests of motor function were conducted concurrently at times when the monkeys were not engaged in cognitive testing. The social separation task was conducted on a single day during which monkeys were not engaged in any other task.

Eight months post collection of the published data from scan 1, both male and female marmosets (7 females, mean age = 6.82 years, SD = 0.63; 8 males, mean age = 6.82 years, SD = 0.88) from that same sample were tested on reversal learning and intradimensional and extra dimensional (ID/ED) set shifting tasks. For correlational purposes, we focused on the cognitive task with the most significant sex difference, the reversal learning task. We investigated the relationship between performance on this task and whole brain connectivity metrics to determine any potential sex differences at scan 2 and effects of time between scans 1 and 2.

### Reversal learning

Male and female marmosets were tested on a series of cognitive tasks as outlined in the LaClair et al. 2019 study^9^. These tasks included the Simple Reversal Learning (SRL) task, a measure of cognitive flexibility, as well as the ID/ED which measures attentional set shifting. A full description of the SRL task can be found in our earlier study. To summarize, this task presents the marmosets with three pairs of stimuli. Within each pair, the marmosets are required to learn the stimulus/reward contingencies to obtain a reward (dehydrated marshmallow). Once 90% accuracy is met for identifying the stimulus that will be rewarded (discrimination), the stimulus/reward contingencies are reversed (reversal). The monkey has to perform the reversal until a 90% learning criterion, after which a new stimulus pair of stimuli is presented. The main dependent variable is the Reversal Index (RI), which is the ratio of the mean number of trials for the marmoset to reach the 90% learning criterion on the 3 reversals by the mean number of trials for the marmoset to reach the 90% learning criterion on the 3 discriminations.

### Imaging

Following our work in LeClair et al. 2019, we used the same state-of-the-art technique developed by Dr. Afonso Silva^66^ to image awake marmosets without the use of anesthetic. Each animal wore a sleeveless jacket (Lomir Biomedical, Inc) which attached to a semi-cylindrical plastic cover made of Lexan, restricting anterior or posterior movement but allowing the animal to move its arms, legs, and tail freely. The plastic cover was attached to the back of the marmoset’s jacket using plastic cable ties. The monkey lay in a supine sphinx position in the MRI bed, which consisted of a 111-mm cylindrical tube. The cover was secured to the bed by screwing nylon thumb screws into the bars on the bed. Each marmoset wore an individualized helmet adapted to their skull to support the head and prevent movement while providing comfort.

### Acclimation

Prior to imaging sessions, animals were acclimated to the bed restraint device, noise related to imaging, and the helmet, following the procedures detailed in Silva et al. 2011^66^. The entire acclimation period took 4–6 weeks for each animal, with acclimation occurring 4–5 days a week.

### fMRI data acquisition

The monkeys underwent MRI at the Center for Comparative Neuroimaging at the University of Massachusetts Medical School. Following 1 h acclimation to the neuroimaging room, marmosets were placed in jackets, positioned in the MR bed, and imaged using a custom head coil as described in Silva et al. 2011^66^. Imaging was carried out on a high-field Bruker Biospin MRI system. The system includes a 4.7 T/40 cm horizontal magnet (Oxford, UK) equipped with 450 mT/m magnetic field gradients and a 20-G/cm magnetic field gradient insert (inner diameter = 11.5 cm; Bruker, Germany) with a digital interface to Bruker console, run by Paravision 6. Field map measurements allowed the estimation of the magnetic field inhomogeneity and shimming. For each marmoset, anatomical images were obtained using rapid acquisition relaxation enhanced (T2 Turbo RARE) sequence with TR (relaxation time) = 2892.97 ms, RARE factor = 8, TE (echo time) = 36 ms, resolution matrix = 256 × 256, FOV (field of view) = 45 mm × 45 mm, slice number = 25, slice thickness = 1.1 mm. Functional images were acquired using echo-planar imaging (EPI) with the same FOV and slice thickness, TR = 1691.04 ms, TE = 26.52 ms, flip angle = 90°, and resolution matrix = 128 × 128, for 11.27 min (400 repetitions). All monkeys were scanned within the period of their cognitive testing.

### Resting state functional connectivity image processing

Brain masks were generated using FMRIB Software Library’s (FSL) Brain Extraction Tool (BET)^67^ on anatomical scans and masks were then manually adjusted with the help of ITK-SNAP (https://www.itksnap.org). The masks outlining the brain were used to remove non-brain voxels. N4 bias field correction^68^ was used to remove B1 field inhomogeneities and improve anatomical image quality prior to alignment. The cropped anatomical brain images were aligned with a Marmoset brain template^69^ using the FSL linear registration program FLIRT^70^. Alignment to the Marmoset template was further optimized using nonlinear symmetric normalization (SyN) in Advanced Normalization Tools (ANTs)^71^. Linear and nonlinear registration matrices for each subject were saved and used to subsequently transform functional datasets into atlas space for preprocessing and analysis. Aside from Subject-to-Atlas registration, which used FSL FLIRT, post processing steps were carried out using Analysis of Functional NeuroImages (AFNI)^72^. AFNI’s 3dDespike was used to remove time series spikes and this was followed by slice timing correction using 3dTshift.

Motion correction was carried out using 3dvolreg, after which functional scans were aligned with the Marmoset template using FLIRT and ANTs. Time series from motion estimates and from areas with cerebrospinal fluid (CSF ventricles) and white matter were used as regressors. AFNI’s 3dTproject was used for the removal of motion-related, CSF and white matter signals, spatial blurring (0.8 mm FWHM), and whole brain voxel-wise bandpass filtering between 0.01–0.1 Hz. 3dmaskSVD was used to extract the principal singular vector time series of each region of interest (ROI) based on the atlas-guided seed location (122 bilateral placed seed regions included for 244 total ROI). Time series were normalized such that the sum of its time points squared was 1. These were exported as individual text files per ROI and used voxel-wise cross correlations were carried out to create correlation coefficient (Pearson r) maps using AFNI 3dTcorr1D^73^. Composite functional connectivity maps were generated using AFNI 3dTtest++ for cortical and subcortical seed regions to determine differences between male and female marmosets (p < 0.01). In addition, 1dCorrelate in AFNI was used to compute Pearson r coefficients for all ROI time series pairs. The AFNI 1dCorrelate tabulated output with a total of 29,646 r values was imported into MATLAB, values z-transformed, and organized into symmetric matrices for network analysis (see below).

### Network analyses

Basic graph theory algorithms available in Brain Connectivity Toolbox for MATLAB^74^ were used to assess the topology of the functional connectivity networks. Symmetrical connectivity graphs with a total 29,646 matrix entries were first organized in MATLAB (graph size = n(n − 1)/2, where n is the number of nodes represented in the graph, or 244 ROI). The z-score values of the graphs were thresholded at various levels (1–40%) to preserve an equal density of the top functional connectivity correlation values per graph prior to network metric assessments. Matrix z-values were normalized by the highest z-score, such that all matrices had edge weight values ranging from 0 to 1. Node strength (sum of edge weights), clustering coefficient (the degree to which nodes cluster together in groups), average shortest path length (the potential for communication between pairs of structures), modularity (the degree to which the network may be subdivided into clearly delineated groups or communities), and small worldness (the degree to which functional brain networks deviate from randomly connected networks) were calculated for weighted or unweighted graphs^75–79^.

The small world coefficient was determined by comparing marmoset functional connectivity networks to a null network generated in brain connectivity toolbox^74^. This step involved subjecting each original functional connectivity graph (weighted undirected graphs) to a randomization process that preserves degree and strength values of the original graph. Each edge was sorted an average of 10 times during the functional connectivity graph randomization. Thus, the ratio for clustering coefficients and path lengths of marmoset brain relative to null network was calculated. The ratio of clustering coefficients is known as γ, which for a small world network is larger than 1^79^. The ratio of average path length is referred to as λ, which for a small world network is close to 1. The small world (sw) parameter is the ratio of γ/λ, with a sw > 1 indicative of small world topology (typical of real world networks) and sw ~ 1 indicative of a random network^80^. Brain networks were visualized using BrainNet Viewer^81^. The 3D networks were generated with undirected edges weights Eundir ≥ 0.15. In these brain networks (or marmoset brain connectomes), the size and color of spheres representing nodes were scaled by node strength, and lines representing connections between nodes were scaled by z-scores. Statistically significant differences were defined as p ≤ 0.05.

## Supplementary information

Supplementary Figure 1.

## Data Availability

The datasets generated during and/or analyzed during the current study are available from the corresponding author on reasonable request.
