# Supplementary material for: Robustness of sex-differences in functional connectivity over time in middle-aged marmosets
Source: Sci Rep. 2020 Oct 6;10:16647. doi: 10.1038/s41598-020-73811-9 (PMC7538565; doi:10.1038/s41598-020-73811-9)
Supplement: Supplementary file 1 — Supplementary Figure 1. [file 41598_2020_73811_MOESM1_ESM.docx]

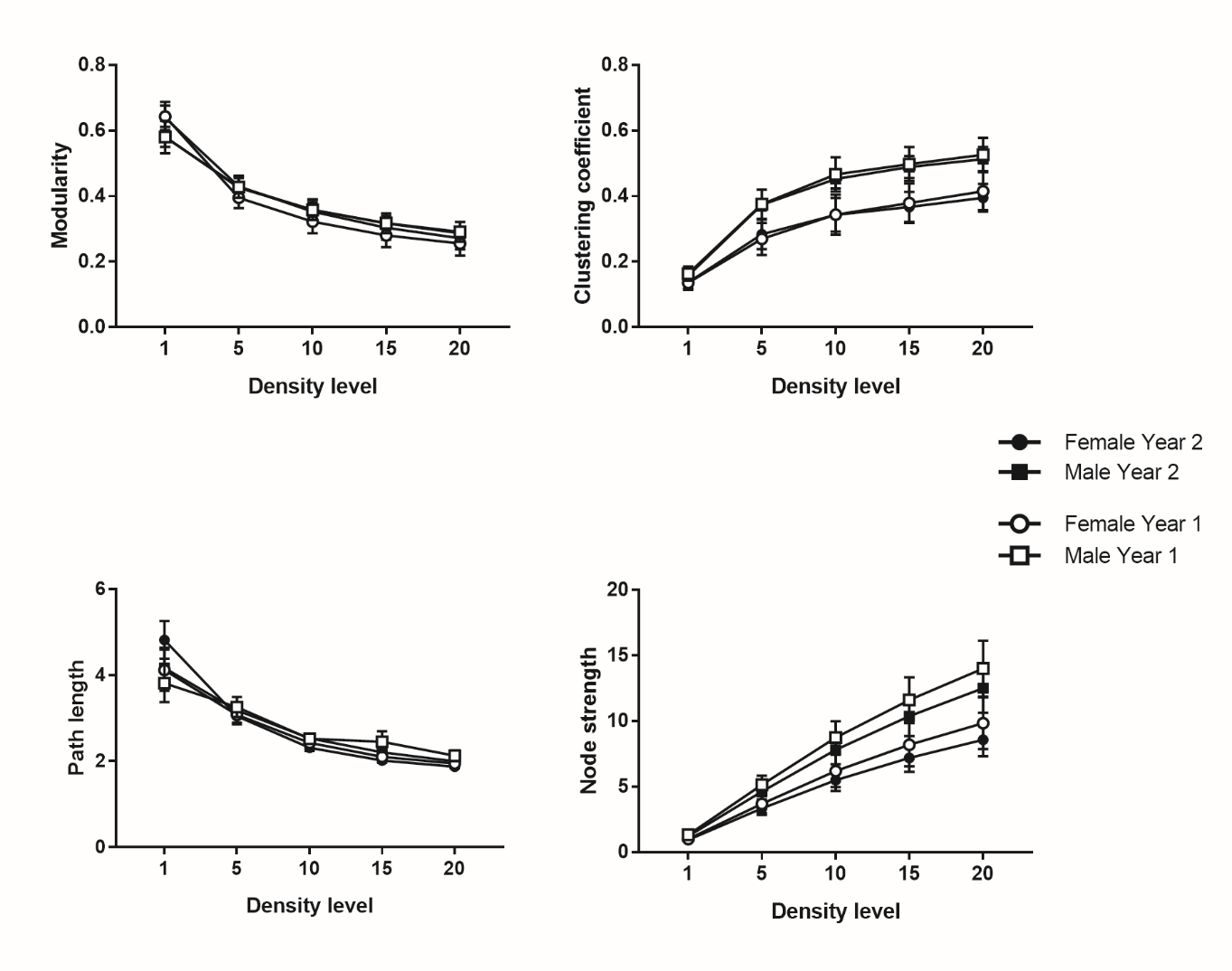


**Supplemental Figure 1** No differences in network measures across scans 1 and 2 in male and female marmosets.
